# Role of postoperative lubrication in preventing dry eye after cataract surgery in high- and low-risk patients stratified by ocular surface frailty index

**DOI:** 10.1371/journal.pone.0312712

**Published:** 2025-03-26

**Authors:** Stefan Palkovits, Andreas Schlatter, Manuel Ruiss, Andreea Fisus, Natascha Bayer, Paul Kofler, Oliver Findl

**Affiliations:** VIROS—Vienna Institute for Research in Ocular Surgery, A Karl Landsteiner Institute, Hanusch Hospital, Department of Ophthalmology, Vienna, Austria; Medizinische Universitat Graz, AUSTRIA

## Abstract

**Purpose:**

Symptoms and signs of dry eye can significantly increase after cataract surgery. The present study seeks to investigate the effect of intensive lubrication using Systane HYDRATION multi-dose preservative free (MDPF) eye drops (Alcon Research, Ltd., Fort Worth, Texas, USA) on signs and symptoms of dry eye in the postoperative phase.

**Methods:**

Patients scheduled for cataract surgery were enrolled in this randomised, investigator-masked study and assigned to one of three groups. The risk of developing dry eye was stratified using the ocular surface frailty index (OSFI). The high risk – standard of care (HR-SOC) and treatment (HR-Treatment) group were the high-risk groups (OSFI 0.3 or greater), and the LR-SOC group was the low-risk group (OSFI less than 0.3). HR-SOC and LR-SOC group received the standard postoperative treatment. In the HR-Treatment group, Systane HYDRATION MDPF was added four times a day for three months after surgery. After the baseline examination prior to cataract surgery, three follow up visits were scheduled one week, one month and three months after surgery. OSDI scores, ocular surface staining, and fluorescein break-up time were assessed at each visit.

**Results:**

Ninety-five patients were included and 83 entered statistical analyses. OSDI increased in all groups after cataract surgery, and it was lowest in the HR-Treatment group three months after the surgery. In addition, fluorescein break-up time tended to be longer and ocular surface staining less in the HR treatment group at three months. However, there was no statistically significant difference between the groups during the three-month visit.

**Conclusion:**

In this randomized, examiner-masked trial no statistically significant difference could be found between the groups, but Systane HYDRATION MDPF may have a positive effect after cataract surgery in high-risk groups for dry eye syndrome.

**Trial registration:**

ClinicalTrials.gov NCT06555224

## 1. Introduction

Cataract and dry eye are two conditions occurring frequently in the elderly. Several reports suggested an exacerbation of dry eye after cataract surgery, causing a significant impact on patients’ postoperative satisfaction as well as quality of life up to six months after surgery [1]. This condition was named “surgically induced iatrogenic dry eye disease” by the Tear Film and Ocular Surface Society International Dry Eye Workshop II [2].

It is believed that the onset or exacerbation of dry eye after cataract surgery is a multifactorial process, leading to tear film disturbances post-operatively. Different causes were published so far, such as reduction of corneal sensitivity due to transection of the corneal nerves, forceful opening of the eyelids, age, long-lasting exposure to light, elevation of inflammatory factors in the tear film, topical anesthesia or povidone-iodine instilled before surgery, as well as pre and/or post-surgical medication and preservatives [2–7].

Recently, a novel index, named the ocular surface frailty index (OSFI), for assessing the risk of developing dry eye after cataract surgery in a non-dry eye population, was published [8]. This score utilizes 10 items including clinical ocular findings, medical history as well as environmental factors and grades the respective subject into a low-risk- and high-risk group. Using the cut-off of 0.3, the rate of postoperative dry eye was 9.6% in the low-risk group and 50% in the high-risk group.

Systane HYDRATION multi-dose preservative free (MDPF) eye drops (Alcon Research, Ltd., Fort Worth, Texas, USA) are an artificial tear substitute based on the dual-polymer formula containing hydroxypropyl (HP)-Guar and hyaluronic acid (HA). Previous studies found an increase in tear film stability and reduction of subjective complaints after the treatment using this compound [9]. In an animal corneal abrasion model, corneas treated with Systane HYDRATION showed the fastest re-epithelialization compared to other HA products supporting the beneficial role of HA-containing artificial tears in corneal wound healing [10]. In a retrospective, multicenter cohort study, the application of drops containing HP-Guar and HA significantly reduced signs and symptoms of dry eye after cataract surgery up to 8 weeks after cataract surgery [11].

The current study investigated whether intensive lubrication with Systane HYDRATION MDPF in the postoperative phase of cataract surgery can reduce the symptoms and signs of dry eye after cataract surgery, in patients with a high-risk OSFI score as compared to a low-risk group.

### 1.1. Hypothesis

Postoperatively administered lubrication eye drops reduce the signs and symptoms of dry eye after cataract extraction in patients with high-risk (OSFI score ≥ 0.3) to develop dry eye symptoms compared to standard of care and the LR-SOC group (OSFI score < 0.3).

## 2. Methods

Prior to the start of the study, all study-relevant documents were reviewed and approved by the Ethics Committee of the city of Vienna (EC number: EK21-098-0521). The study was conducted in accordance with the Declaration of Helsinki and its subsequent amendments and the European Union guidelines for good clinical practice. The study was registered at www.clinicaltrials.gov (Identifier: NCT06555224).

### 2.1. Patients

Patients scheduled for cataract surgery were included in the study and were selected by the clinical investigators at the Department of Ophthalmology of the Hanusch Hospital, Vienna. Inclusion criteria were age over 18 years, scheduled cataract surgery using a monofocal or monofocal-toric IOL and a preoperative modified ocular surface disease index (OSDI) score (see 2.5.) lower than 13. Patients were assigned to one of three groups according to their OSFI score. Patients with OSFI greater or equal to 0.3 were randomized into high risk - standard of care (HR-SOC) or treatment group (HR-Treatment), using an online randomization tool (https://randomizer.org, list randomizer) by an unmasked member of the study staff. Patients with OSFI lower than 0.3 were included in the LR-SOC group. The presence of ocular symptoms of dry eye preoperatively and intraoperative complications excluded the patient from the study. Usage of glaucoma medication did not exclude the patient from study participation. Further criteria excluding the patients were other ocular surgery, infection or injury in the past three months, active ocular inflammation or infection, pregnancy, or severe lid abnormalities.

### 2.2. Study design

All patients underwent a screening examination after giving their written informed consent. After successful screening, four study visits were scheduled – baseline within 30 days prior to cataract surgery, one week (7 ± 1 days), one month (30 ± 7 days) and three months (90 ± 7 days) after cataract surgery. Occurrence of dry eye signs and symptoms were evaluated during each study visit and the following measurements were performed by a masked investigator: OSDI score, fluorescein staining/ lissamine green staining scores and fluorescein break-up time (FBUT).

### 2.3. Group allocation

Patients in the HR-SOC group received routine postoperative treatment using bromfenac (Yellox, Bausch and Lomb, Germany) eye drops twice a day for four weeks after surgery and served as the standard of care group. Patients in the HR-Treatment group used Systane HYDRATION MDPF eye drops in both eyes four times a day starting after surgery until the three-month visit in addition to the routine treatment with bromfenac. Patients with OSFI scores below 0.3 (low risk) were included in the LR-SOC group and received the routine treatment using bromfenac twice a day for four weeks. During the study period no other drops (such as topical antibiotics or steroids) were used.

Summary of groups and treatments:

- **High risk: Standard of care group (HR-SOC)** OSFI ≥  0.3, bromfenac eye drops (2x/day for 4 weeks)
- **High risk: Treatment group (HR-Treatment)** OSFI ≥  0.3, Systane HYDRATION MDPF eye drops (4x/day for 3 months), bromfenac eye drops (2x/day for 4 weeks)
- **Low risk: Standard of care group (LR-SOC)** OSFI <  0.3, bromfenac eye drops (2x/day for 4 weeks)

All patients were asked not to use any other eye drops or ointments during the study period.

### 2.4. Masking

The dry eye assessments (corneal and conjunctival staining, FBUT) were carried out by a masked examiner who did not know the respective group allocation. Group allocation and randomization were carried out by unmasked study personnel.

### 2.5. Ocular Surface Disease Index (OSDI)

Dry eye symptoms were evaluated using the OSDI score, as established by Schiffman et al. in 2000 [12]. In general, this scoring system comprises a set of 12 items regarding the frequency of symptoms and their effect on vision-related activities over the preceding week and factors influencing the symptoms. Patients rated them from “none of the time” to “all the time”. The OSDI score is calculated by multiplying the sum of all items scores with 25 and dividing it by the number of questions responded to. In this study, a modified OSDI score was applied, in which only question 2, 3, 10, 11 and 12, directly related to dry eye symptoms, were considered. Questions regarding visual function were analyzed separately. (1, 4, 5, 6, 7, 8, 9).

### 2.6. Assessment of ocular surface

Ocular surface assessment included fluorescein corneal staining, lissamine green staining and assessment of FBUT. The investigator graded corneal staining in accordance with the National-Eye-Institute (NEI) grading scale, where the cornea is separated into five zones, within each zone the amount of staining was graded between 0 and 3 [13]. Therefore, a total score of 0-15 could be reached. For grading of lissamine green staining modified Oxford scale was applied. Staining of the temporal and nasal conjunctiva and the cornea was evaluated on a scale of 0 to 4. Therefore, a total score of 0-12 could be reached. Both values (corneal and conjunctival staining) were then added together, and the total staining score was calculated.

The FBUT was measured after the instillation of 1 µl of fluorescein into the inferior fornix. Patients were asked to blink several times to spread the fluorescein on the ocular surface. A stopwatch was used to measure the time from the last blink to the first tear film break-up. Three consecutive measurements were taken, and the average was calculated.

### 2.7. Cataract surgery

Routine cataract surgery was performed under topical anesthesia. The procedure included a 2.4 mm incision, injection of an ophthalmic viscosurgical device (OVD), performance of capsulorhexis, phacoemulsification and coaxial irrigation/aspiration of cortical material. After implantation of the intraocular lens and removal of the OVD, the incision was routinely hydrated to seal the wound.

In the preoperative phase, the following eye drops were administered to ensure adequate pupil dilation: cyclopentolate 1% (“Thilo”, Alcon Ophthalmika GmbH) and tropicamide 1%/phenylephrine 2.5% (Hanusch Hospital pharmacy, non-preserved), each given four times. For local anesthesia, oxybuprocaine 0.4% (Novesin 0.4%, Omnivision) eye drops were used twice.

Just before surgery, povidone-iodine eye drops (0.9%), as well as povidone-iodine skin solution were applied twice for disinfection.

### 2.8. Objectives and statistics

The primary objective was to assess the subjective symptoms determined using the modified OSDI score after cataract surgery over the study period and between the groups. Secondary objectives were to compare the signs of dry eye (total staining score, FBUT) during the study period.

SPSS (version 29.0) was used for the statistical analysis. Descriptive data are presented as mean, standard deviation and range. Differences in baseline study characteristics (age, OSFI score) were evaluated using an ANOVA between groups. We used linear mixed effects models to test the hypotheses of a difference in the dry eye parameters (total staining score, FBUT) and modified OSDI between the study groups throughout the study. We used the respective outcome as dependent variable and added fixed effects of study group, study day and the interaction of study group and study day. Patients were added as a random effect. The model specification was as follows:

outcome ~  ‘study day’ +  ‘study group’ +  ‘study day * ’study group’ +  (1 | patientID)

As an exploratory analysis a linear mixed model was created to assess the score of each OSDI item in the whole study population. In that regard, the baseline and three-month visit were considered for statistical analysis, and the following formula was used:

outcome ~  ‘study day’ +  ‘OSDI item’ +  ‘study day * OSDI item’ +  (1 | patientID)

Bonferroni post-hoc analysis was used for group-wise comparison in all models. P-values were obtained using maximum likelihood ratio tests. A p-value of < 0.05 was considered significant.

### 2.9. Patient number and sample size calculation

For sample size calculations we used the data published earlier by Villani et al. [8]. As group allocation was based on OSFI score, we used the previous data on postoperative dry eye rates (high risk: 50%; low risk 9.6%) to calculate the sample size and to detect differences between the groups. In that regard and using an alpha error of 0.05 and a power of 80% we planned to include 117 patients, including a dropout rate of 30%.

## 3. Results

### 3.1. Study population

**Fig 1:**
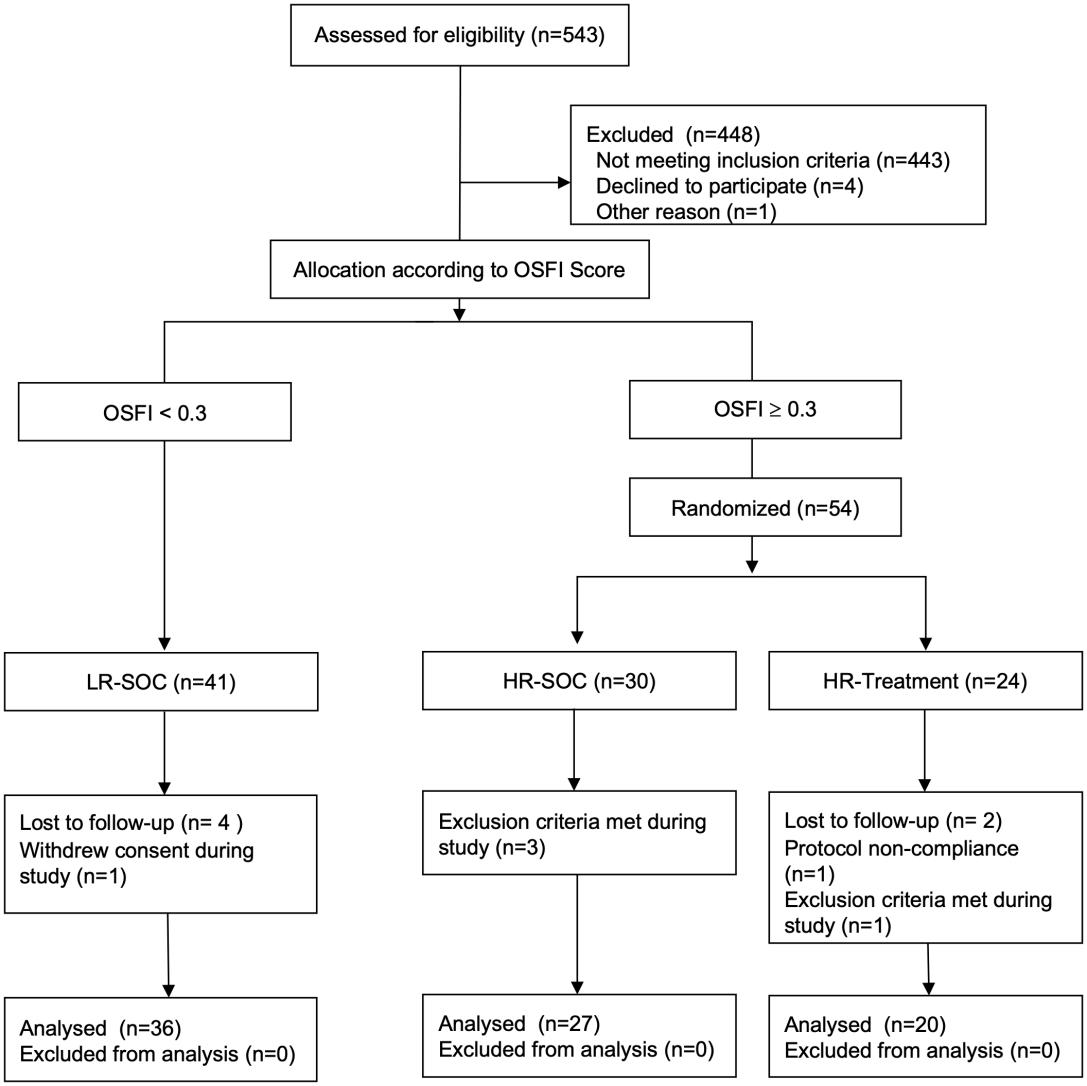
CONSORT Flow diagram; OSFI, Ocular surface frailty index; HR-SOC, high risk - standard of care; LR-SOC, low risk - standard of care.

In total, 543 patients were screened prior to cataract surgery. Ninety-five patients were finally enrolled in this study, 83 of whom were included in the statistical analyses. Twelve patients were excluded for the following reasons: six patients did not attend follow-up visits as scheduled, one patient withdrew their consent during the study, four patients were excluded due to intraoperative or postoperative complications (one patient with a postoperative corneal erosion, two patients with a capsule rupture, one patient with pronounced postoperative inflammation), and one patient did not take the study medication as intended. Consort flow diagram is shown in Fig 1. Recruitment started on the 9^th^ of August 2021 and the last patient was enrolled on 20^th^ of September 2023. Table 1 summarizes the characteristics of the study population.

**Table 1:** Study characteristics.

	HR-SOC	HR-Treatment	LR-SOC	p
	n = 27	n = 20	n = 36	
**Age** (years)	72 ± 7.6	71 ± 8.5	70 ± 8.1	0.683
	(56 to 85)	(54 to 85)	(54 to 82)	
**Female/Male**	11/9	9/9	19/11	
**OSFI**	0.36 ± 0.06	0.37 ± 0.05	0.19 ± 0.07	**<0.001**
	(0.30 to 0.53)^*^	(0.30 to 0.46)^*^	(0 to 0.29)	

Data are presented as mean±SD (range);

^a^ANOVA, groupwise comparison using Bonferroni correction,

* indicates significant difference to LR-SOC group; OSFI, ocular surface frailty index; HR-SOC, high risk - standard of care; LR-SOC, low risk - standard of care.

### 3.2. OSDI scores

**Fig 2:**
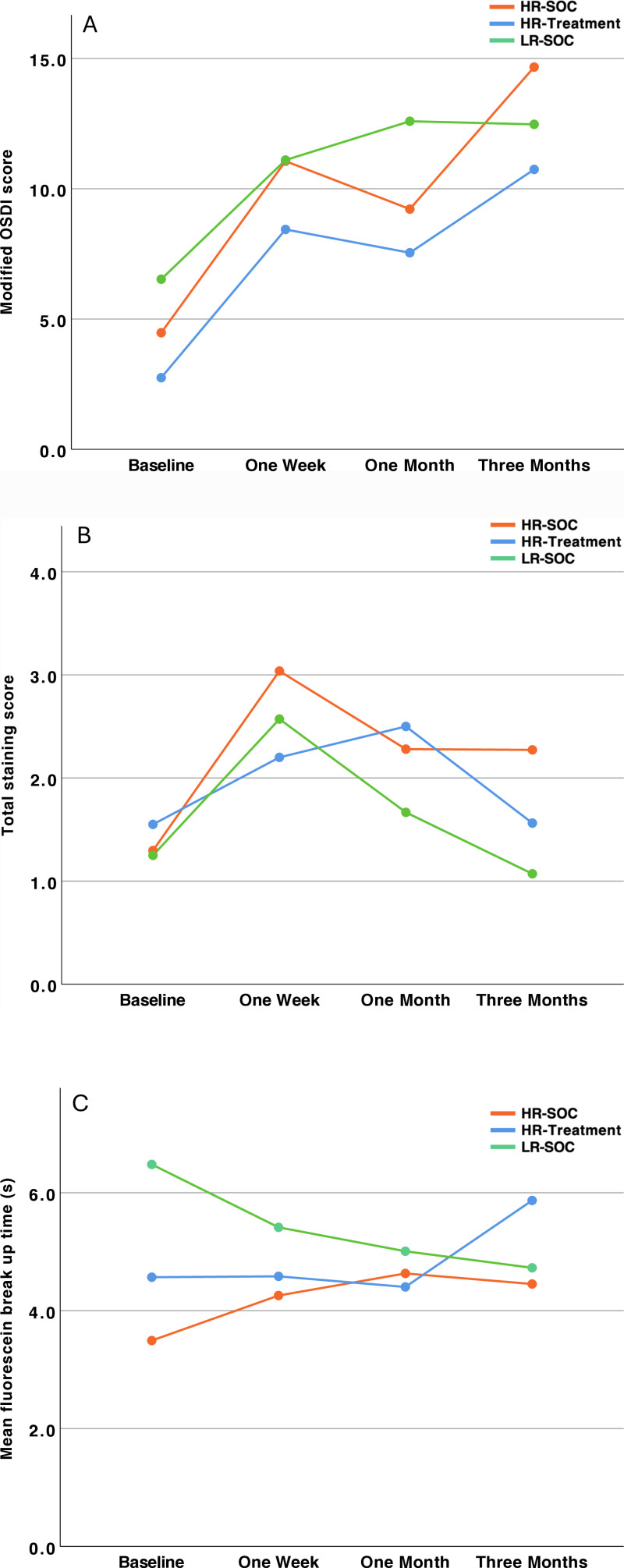
A: Mean modified OSDI score; B: Total staining score; C: Mean fluorescein break-up time; HR-SOC, high risk - standard of care; LR-SOC, low risk - standard of care. OSDI, Ocular surface disease index.

In the linear mixed model, there was a significant main effect of ‘time’ on the modified OSDI value (p < 0.001), but not for ‘group’ (p = 0.435) or the interaction term (p = 0.931). Modified OSDI increased in all groups and reached statistical significance for the HR-SOC group after three months (p = 0.01). At this point, OSDI scores were lowest in the HR treatment group and highest in the HR-SOC group, but there was no statistically significant difference between the groups. Fig 2A shows the mean modified OSDI scores over the study period for all groups. Results for groupwise comparison is presented in Table 2.

**Table 2:** Linear mixed model results for modified OSDI.

Modified OSDI							Difference in modified OSDI					
	HR-SOC		HR-Treatment		LR-SOC		HR-SOC - HR-Treatment		HR-SOC - LR-SOC		LR-SOC - HR-Treatment	
	n	mean	n	mean	n	mean	mean	p	mean	p	mean	p
		(95% CI)		(95% CI)		(95% CI)	(95% CI)		(95% CI)		(95% CI)	
**Baseline**	27	4.50	20	2.80	36	6.50	−1.73	1.0	−2.05	1.0	3.78	0.87
		(−0.4 to 9.3)		(−2.9 to 8.4)		(2.3 to 10.7)	(−7.34 to 10.8)		(−9.88 to 5.77)		(−4.8 to 12.35)	
**One Week**	26	11.10	20	8.40	36	11.10	−2.64	1.0	−0.03	1.0	2.66	1.0
		(6.2 to 16.0)		(2.8 to 14.1)		(6.9 to 15.3)	(−6.49 to 11.76)		(−7.92 to 7.87)		(−5.9 to 11.24)	
**One Month**	24	9.30	19	7.60	33	12.90	1.69	1.0	−3.63	0.85	5.33	0.44
		(4.2 to 14.3)		(1.83 to 13.3)		(8.6 to 17.2)	(−7.67 to 11.06)		(−11.8 to 4.53)		(−3.47 to 11.12)	
**Three Months**	20	14.20	18	11.40	28	13.10	−2 2.82	1.0	−1.08	1.0	1.74	1.0
		(8.7 to 19.6)		(5.5 to 17.2)		(8.5 to 17.7)	(−6.97 to 12.61)		(−7.67 to 9.83)		(−7.39 to 10.87)	

p-value for pairwise comparison (‘group’) – corrected using Bonferroni correction; OSDI, Ocular surface disease index; FBUT, Fluorescein break-up time; HR-SOC, high risk - standard of care; LR-SOC, low risk - standard of care.

### 3.3. Dry eye parameters

We observed a significant effect of time (p < 0.001), but not for group or the interaction term (p = 0.356 and 0.354 respectively) on the total staining scores. An increase was observed one week following the procedure in all groups. This observed increase was found to be statistically significant in both the HR-SOC and LR-SOC groups (p = 0.002 and p = 0.011 respectively). No statistically significant increase was observed in the HR-Treatment group. After three months, the highest staining scores were observed in the HR-SOC group. No statistically significant difference was found between the groups over the study period. Fig 2B shows a summary of the total staining scores and Table 3 the results for the group wise comparisons.

**Table 3:** Linear mixed model results for total staining score.

Total staining score							Difference in total staining score					
	HR-SOC		HR-Treatment		LR-SOC		HR-SOC - HR-Treatment		HR-SOC - LR-SOC		LR-SOC - HR-Treatment	
	n	mean	n	mean	n	mean	mean	p	mean	p	mean	p
		(95% CI)		(95% CI)		(95% CI)	(95% CI)		(95% CI)		(95% CI)	
**Baseline**	27	1.30	20	1.60	36	1.30	−0.3	1.0	0.0	1.0	0.3	1.0
		(0.5 to 1.1)		(0.6 to 2.5)		(0.5 to 2.0)	(−1.8 to 1.3)		(−1.3 to 1.4)		(c1.8 to 1.2)	
**One Week**	27	3.00	20	2.20	36	2.60	0.8	0.57	0.5	1.0	0.4	1.0
		(2.2 to 3.9)		(1.2 to 3.2)		(1.9 to 3.3)	(−0.7 to 2.4)		(−0.9 to 1.8)		(−1.1 to 1.8)	
**One Month**	24	2.20	19	2.50	33	1.70	−-0.3	1.0	0.6	0.91	-0.9	0.53
		(1.4 to 3.1)		(1.5 to 3.5)		(0.9 to 2.4)	(−1.9 to 11.3)		(−0.8 to 2.0)		(−2.4 to 0.7)	
**Three Months**	20	2.30	18	1.60	28	1.00	0.7	0.92	1.3	0.1	-0.6	1.0
		(1.4 to 3.2)		(0.5 to 2.6)		(0.2 to 1.8)	(−1.0 to 2.4)		(−0.2 to 2.7)		(−2.2 to 1.0)	

p-value for pairwise comparison (‘group’) – corrected using Bonferroni correction; HR-SOC, high risk - standard of care; LR-SOC, low risk - standard of care.

Fluorescein break-up time was significantly higher in the LR-SOC group as compared to the other groups during baseline, which constitutes a component of the inclusion criteria (OSFI score) and, is therefore, anticipated. A significant reduction in break-up time was observed for the LR-SOC group over the course of the study (baseline – three months: -1.74s; p = 0.03). On the other hand, in the HR-SOC as well as the HR-Treatment group an increase in break-up time could be found, which was however not statistically significant. Fig 2C shows the FBUT during the study period. Table 4 shows results of the linear mixed model analyses for FBUT.

**Table 4:** Linear mixed model results for FBUT.

FBUT							Difference in FBUT					
	HR-SOC		HR-Treatment		LR-SOC		HR-SOC - HR-Treatment		HR-SOC - LR-SOC		LR-SOC - HR-Treatment	
	n	mean	n	mean	n	mean	mean	p	mean	p	mean	p
		(95% CI)		(95% CI)		(95% CI)	(95% CI)		(95% CI)		(95% CI)	
**Baseline**	27	3.50	20	4.60	36	6.50	−1.07	0.68	−2.99	**<0.001**	1.91	0.07
		(2.4 to 4.6)		(3.2 to 5.9)		(5.5 to 7.5)	(−3.22 to 1.07)		(−4.84 to-1.14)		(−0.11 to 3.94)	
**One Week**	26	4.30	20	4.60	36	5.40	−0.33	1.0	−1.17	0.4	0.83	0.97
		(3.1 to 5.4)		(3.3 to 5.9)		(4.4 to 6.4)	(−2.49 to 1.83)		(−3.03 to 0.70)		(−1.19 to 2.86)	
**One Month**	24	4.70	19	4.40	33	5.00	0.28	1.0	−0.33	1.0	0.62	1.0
		(3.5 to 5.9)		(3.0 to 5.7)		(4.0 to 6.0)	(−1.94 to2.51)		(−2.27 to 1.61)		(−1.47 to 2.70)	
**Three Months**	20	4.50	18	5.80	28	4.70	-1.34	0.51	−0.23	1.0	−1.11	0.66
		(3.2 to 5.8)		(4.4 to 7.2)		(3.6 to 5.8)	(−3.69 to 1.00)		(−2.33 to 1.88)		(−3.30 to 1.07)	

p-value for pairwise comparison (‘group’) – corrected using Bonferroni correction; FBUT, Fluorescein break-up time; HR-SOC, high risk - standard of care; LR-SOC, low risk - standard of care.

### 3.4. Exploratory analysis of each OSDI item

**Fig 3:**
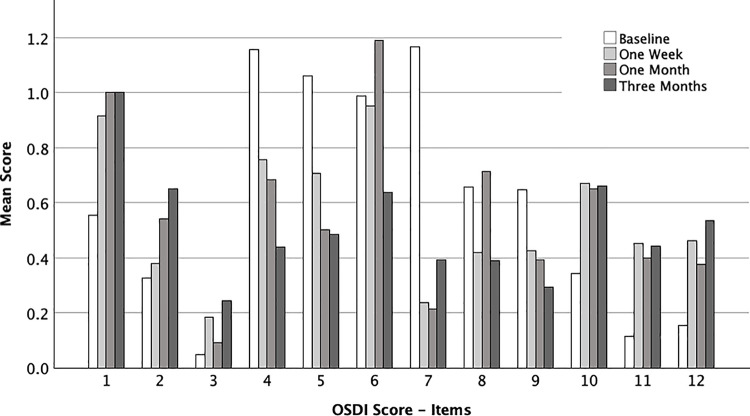
Mean score for each OSDI item; OSDI, Ocular surface disease index.

Table 5 summarizes the results for each OSDI item during baseline and the three months visit. OSDI item 1, 2, 10, 11, and 12 was significantly higher at the three months visits, whereas item 4, 5, 6, 7, and 9 declined significantly. Fig 3 shows the course of the individual OSDI items over the study period.

**Table 5:** Linear mixed model OSDI items.

OSDI item	Baseline	Three Months	Difference in item score	
			Three Months - Baseline	
	Mean	Mean	Mean	p
	(95% CI)	(95% CI)	(95% CI)	
1	0.58	1.05	0.47	<.001
	(0.37 to 0.79)	(0.83 to 1.28)	(0.2 to 0.75)	
2	0.35	0.71	0.36	0.012
	(0.14 to 0.56)	(0.48 to 0.93)	(0.08 to 0.63)	
3	0.07	0.3	0.22	0.116
	(−0.13 to 0.28)	(0.07 to 0.52)	(−0.06 to 0.63)	
4	1.18	0.49	−0.69	<.001
	(0.97 to 1.39)	(0.26 to 0.72)	(−0.97 to − 0.41)	
5	1.09	0.53	−0.56	<.001
	(0.88 to 1.3)	(0.3 to 0.76)	(−0.85 to − 0.28)	
6	1.01	0.69	−0.32	0.027
	(0.8 to 1.22)	(0.46 to 0.92)	(−0.6 to − 0.04)	
7	1.15	0.41	−0.74	<.001
	(0.93 to 1.28)	(0.14 to 0.68)	(−1.07 to − 0.42)	
8	0.67	0.43	−0.25	0.096
	(0.46 to 0.89)	(0.19 to 0.67)	(−0.54 to 0.05)	
9	0.67	0.34	−0.328	0.022
	(0.46 to 0.88)	(0.11 to 0.57)	(−0.61 to − 0.05)	
10	0.37	0.71	0.339	0.018
	(0.17 to 0.58)	(0.48 to 0.94)	(0.06 to 0.62)	
11	0.15	0.52	0.37	0.011
	(−0.06 to 0.36)	(0.29 to 0.76)	(0.08 to 0.66)	
12	0.19	0.61	0.42	0.005
	(−0.02 to 0.4)	(0.37 to 0.85)	(0.13 to 0.71)	

p-value for pairwise comparison (‘Study day’) – corrected using Bonferroni correction; OSDI, Ocular surface disease index.

## 4. Discussion

This study investigated the influence of postoperative lubrication on dry eye parameters in patients after cataract surgery. We found that postoperative dry eye symptoms and signs were not significant different three months after the procedure between the groups with a frail ocular surface and in patients with a more robust ocular surface, as judged using the previously published OSFI score. However, one week after surgery, the SOC groups without additional lubrication showed a significant increase in the total staining score, whereas it was not significant in the treatment group. This finding reflects a positive effect of postoperative lubrication but could not completely prevent the occurrence of staining of the ocular surface. At the end of the study period, the HR-SOC group had the highest staining score, followed by the HR-treatment and LR-SOC groups, respectively, although this difference was not statistically significant.

This is in good agreement with the marked increase in subjective complaints using the modified OSDI score after one week, which was highest in the HR-SOC group at the end of the study period. As expected, we found the lowest score in the HR-Treatment group at this time, but again this difference was not statistically significant and could only be interpreted as a trend. Interestingly, the modified OSDI scores were also higher in the LR-SOC group than in the HR-Treatment group, although the latter group had a higher OSFI score. After the initial increase in subjective complaints at one week, it remained almost stable in the LR-SOC group and increased slightly in the HR-Treatment group throughout the rest of the study. In the HR-SOC group, this increase was more pronounced after three months than in the other groups. This suggests that high-risk patients are more likely to develop more severe symptoms and may reflect the beneficial effects of postoperative lubrication in reducing this risk. However, these results must again be interpreted with caution, as we could only observe trends that were not statistically significant.

The FBUT was significantly higher in the LR-SOC group at the start of the study, which can be attributed to the inclusion criteria (OSFI score). FBUT in the LR-SOC group declined significantly over the study period. Interestingly, the FBUT increased in both risk groups, although the increase was greater in the treatment group, resulting in higher FBUT values at the end of the study period. In the treatment group, this finding can be attributed to the effect of lubrication. However, the explanation for the increase in the HR-SOC group is less clear. A possible explanation could be regression to the mean, as FBUT values were lower at baseline compared to the other groups. This could be compounded by the fact that the reduced FBUT was already so low that the chances of further reduction are also limited.

Overall, interpretation of these results suggests an increase in dry eye signs and symptoms after cataract surgery, which is more pronounced in patients with higher OSFI, which could potentially be reduced with Systane HYDRATION eye drops.

Previous studies evaluated the effect of different compounds on the postoperative dry eye, such as hyaluronic acid/trehalose [14], carbomer sodium hyaluronate trehalose and sodium hyaluronate eye drops [3] diquafosol [15,16], hydroxypropyl guar and hyaluronic acid [11] or cyclosporin 0.05% [17]. Although some studies found an improvement in the postoperative period, this effect was not consistent between all studies. In general, the diagnostic parameters of the ocular surface show high variability and high intra- and interrater variability, making them difficult to study. Therefore, studies with large patient populations and objectifiable outcomes are necessary to detect differences in these parameters.

As in previous studies, ocular symptoms and subjective complaints were judged using the OSDI score. It turned out that one major limitation of the OSDI score is the influence of the present cataract on visual function, which is an integral part of OSDI score. Seven out of 12 items are related to visual function, which is impaired in patients with cataract. Our data indicate that directly dry eye related items deteriorate whereas vision related items improve significantly. To our surprise and to the best of our knowledge this is the first study looking into each item of the OSDI separately in patients prior and after cataract surgery. The current study was not designed to investigate this in detail, and thus further studies are required to elucidate the influence of cataract surgery on the OSDI.

In our population, 24% of patients coming for cataract surgery presented with an OSFI higher than or equal to 0.3, which is comparable to Villani et al. In their study the authors found rates of postoperative dry eye symptoms of 50% in patients with a frail ocular surface and 9% in patients with robust ocular surface [8]. Using our modified OSDI score and the same criteria for dry eye the rates of postoperative dry eye symptoms were higher as in the previous study and interestingly not different between the groups (HR-SOC group: 40%; HR-Treatment group 40%; LR-SOC group: 50%). Therefore, these previous results by Villani et al. could not be reproduced in our study. However, the direct comparison is limited as the modified OSDI score was used, which was significantly increased in all study groups three months after cataract surgery. The effects of this modification in OSDI on the evaluation of the dry eye rate are unknown. On the other hand, the influence of the visual items of the conventional OSDI in previous studies on dry eye rates after cataract surgery is also unclear.

Despite this, OSDI scores have been used in several studies to investigate dry eye symptoms before and after cataract surgery. However, the effect of the visual function items on the final OSDI score is often not addressed. Two previous studies omitted visual function questions altogether or removed items 4 and 5 from the questionnaires [18,19]. Fydanaki et al. found a high correlation between the OSDI score - particularly the visual subcategory - and postoperative visual acuity [20]. For that reason, only five items of the OSDI appear useful in assessing dry eye in the perioperative phase. Further studies are necessary to validate and confirm the results of the modified OSDI score in the perioperative phase of cataract surgery.

A major limitation is the small sample size due to the high number of screening failures and drop-outs. A larger number of patients than expected had to be screened to find suitable patients. A problem was the OSDI score as discussed above in combination with the elevated OSFI score. Due to the variability of dry eye parameters, a larger number of patients is needed to further investigate this issue.

## 5. Conclusion

In this randomized, examiner-masked trial no statistically significant difference in dry eye signs and symptoms could be found between the groups three months after cataract surgery. However, a positive effect of postoperative lubrication was observed, especially in the early postoperative phase. To assess dry eye symptoms, a modified version of the OSDI score, as used in this study, or other dry eye questionnaires that assess only dry eye related symptoms may provide more reliable results in the perioperative period of cataract surgery and should be used instead.

## Supporting information

S1 File Datafile 1. This file contains the study data.
(XLSX)

S2 File Datafile 2. This file contains data for each OSDI item.
(XLSX)

S3 File Study protocol version 2.0. (PDF)

S4 File CatDryEye_CONSORT-2010-checklist. (DOC)

## Abbreviations

FBUT: fluoresceine break up time
HA: hyaluronic acid
HP: hydroxypropyl
MDPF: multi-dose preservative free
NEI: National-Eye-Institute
OSDI: ocular surface disease index
OSFI: ocular surface frailty index
OVD: ophthalmic viscosurgical device
SOC: standard of care
HR: high risk
LR: low risk.
