# Supplementary material for: Role of postoperative lubrication in preventing dry eye after cataract surgery in high- and low-risk patients stratified by ocular surface frailty index
Source: PLoS One. 2025 Mar 26;20(3):e0312712. doi: 10.1371/journal.pone.0312712 (PMC11940489; doi:10.1371/journal.pone.0312712)
Supplement: S3 File — (PDF) [file pone.0312712.s003.pdf]

Role of postoperative lubrication in preventing dry eye after cataract surgery

Short title: “Cataract Dry Eye”

Study protocol, 16.12.2021

Vers. 2.0

PD Dr. Stefan Palkovits, PhD

Dr. Andreas Schlatter

Manuel Ruiß, MSc

Prim. Prof. Dr. Oliver Findl, MBA

Vienna Institute for Research in Ocular Surgery (VIROS)

A Karl Landsteiner Institute

Department of Ophthalmology, Hanusch Hospital, Vienna, Austria

Investigator and Study Co-ordinator:

PD. Dr. Stefan Palkovits, PhD

Hanusch Hospital Vienna

Department of Ophthalmology

Vienna Institute for Research in Ocular Surgery (VIROS)

Heinrich-Collin-Straße 30

1140-Vienna

Austria

spalkovits@gmail.com

1 INTRODUCTION 3

1.1 Background 3

1.2 Risk/benefit assessment 3

2 STUDY OBJECTIVE 4

3 INVESTIGATIONAL PLAN 4

3.1 Design 4

3.2 Selection of study population 5
3.2.1 Number of subjects 5
3.2.2 Pre-study screening 5
3.2.3 Inclusion criteria 5
3.2.4 Exclusion criteria 6

3.3 Study protocol 6
3.3.1 Study Visits 6

3.4 Variables and biometric methods 8
3.4.1 Outcome variables 8
3.4.2 Biometric methods 8

4 METHODS OF EVALUATION 9

4.1 Best-corrected visual acuity 9

4.2 Ocular surface disease index – OSDI 9

4.3 Photography of the anterior segment 9

4.4 OSFI Score from Villani et al.[1] 10

4.5 Slit lamp biomicroscopy 10

4.6 Fluorescein Staining / Lissamin green staining grading using the Oxford Scale 10

4.7 Break Up Time (BUT) 10

5 SCHEDULE OF EVENTS 12

6 TIMELINE 12

7 REFERENCES 13

1 Introduction

1.1 Background

Cataract and dry eye are two conditions occurring frequently in the elderly. Several reports suggested a deterioration of dry eye after cataract surgery, causing a significant impact on patients' postoperative satisfaction as well as quality of life.

Recently, a novel index, assessing the risk of developing dry eye after cataract surgery in a non-dry eye population, was published; the so called – Ocular Surface Frailty Index (OSFI).[1] This score utilizes 10 items including clinical ocular findings, medical history as well as environmental factors and grade the respective subject in low-risk- or high-risk group. Using the cutoff of 0.3, the rate of postoperative dry eye was 9,6% in the low-risk group and 50% in the high-risk group ($p<0.001$).

Systane Hydration MDPF eye drops (Alcon Research, Ltd., Fort, Worth, Texas, USA, a division of Novartis) is an artificial tear substitute based on the dual-polymer formula containing HP-Guar and hyaluronic acid. Previous studies found an increase in tear film stability and reduction of subjective complaints.[2]. In an animal model corneas treated with Systane Hydration showed the fastest re-epithelialization compared to other HA products supporting the beneficial role of HA-containing artificial tears in corneal wound healing.[3]

The current study seeks to investigate if intensive lubrication, using Systane Hydration MDPF, in the postoperative phase of cataract surgery can reduce the rate of dry eye in the high-risk group. The findings will have a great impact how to identify these patients, to administer the adequate therapy, which might hinder development of dry eye related to cataract surgery.

1.2 Risk/benefit assessment

Lubricating eye drops have an excellent safety profile especially when preservative-free formulations are used. By adding lubrication to the treatment regime, the patients are not put to an additional risk. All study relevant procedures are used routinely in our department.

The risk/benefit ratio is acceptable.

Postoperativ verabreichte Lubrication-Augentropfen reduzieren die Rate an Symptomen des trockenen Auges nach Kataraktextraktion bei Patienten mit hohem Risiko (Ocular Surface

Frailty Index ≥0,3), Symptome des trockenen Auges zu entwickeln, im Vergleich zur Standardversorgung.

2 Study objective

Hypothesis

Postoperatively administered lubrication eye drops reduce the rate of dry eye symptoms after cataract extraction in patients with high-risk (Ocular Surface Frailty Index ≥0.3) to develop dry eye symptoms compared to standard of care.

OBJECTIVES:

The aim of this study is to compare the signs and symptoms of dry eye after cataract surgery between groups, patients with preoperatively high risk and low risk, and evaluate if adequate and frequently administered lubrication eye drops reduce the signs and symptoms of dry eye in patients with high risk compared to standard of care. The following 3 groups will be compared:

- **Group 1, high risk: Standard of care** OSFI ≥0.3, NSAID (2x day for 4 weeks)
- **Group 2, high risk: Treatment** OSFI ≥ 0.3, Systane Hydration MDPF eye drops (4 x day for 3 months) NSAID (2x day for 4 weeks)
- **Group 3, low risk: Reference** OSFI < 0.3, NSAID (2x day for 4 weeks)

  - **Primary objective:** To compare the rate of dry eye symptoms between Group 1 and Group 2 at 3 months (after cataract surgery)
  - **Secondary objective:** To compare the rate of dry eye symptoms between Group 1 and Group 2 at 1 week and 1 month after cataract surgery. To compare the rate of dry eye disease (DED, Signs and symptoms) between Group 1 and Group 2 at 1 week, 1 and 3 months after cataract surgery.
- Vergleich der Rate der Symptome des trockenen Auges zwischen Gruppe 1 und Gruppe 2 1 Woche und 1 Monat nach der Kataraktoperation. Vergleich der Rate der Erkrankung des trockenen Auges (DED, Anzeichen und Symptome) zwischen Gruppe 1 und Gruppe 2 1 Woche, 1 und 3 Monate nach der Kataraktoperation.

3 Investigational plan

3.1 Design

Prospective, single-centre, controlled, randomized, observer-masked study

3.2 Selection of study population

Participants will be selected by the clinical investigators at the out-patient clinics of the Department of Ophthalmology of the Hanusch Hospital and the connected health centres of the ÖGK, Vienna.

3.2.1 Number of subjects

For sample size calculation we used the data published earlier by Villani et al.[1] In the study 50% of the patients with an OSFI score higher than 0.3 (high risk group) developed dry during the study period. The rate was 9.6% in the group with an OSFI score lower than 0.3. These two proportions were used to calculate the sample size. We assume a clinical benefit of the lubrication drops if the rate of occurrence of dry eye in the high-risk group is reduced close to the value of the low-risk group (15%).
Using an alpha error of 0.05 and a power of 80 % we need to include 27 in group 1 and group 2. Due to the long study period, we expect a dropout rate of 30%. Therefore, we need to include 39 patients in group 1 and group 2. 39 patients will be included in group 3.
In total 117 patients will be enrolled into the study.

3.2.2 Pre-study screening

The following examination will be carried out in each subject:

- Ophthalmic examination
  - Visual acuity
  - Slit lamp biomicroscopy
    - Tear film break up time
    - Meibomian gland expressibility
    - Lipcof
  - OSFI Score
  - Meibography classification using the Meiboscore
  - Indirect ophthalmoscopy
  - Fundoscopy

3.2.3 Inclusion criteria

- Age older than 18 years
- Cataract surgery scheduled in one or both eyes
- Scheduled for monofocal or monofocal-toric IOL implantation
- OSDI lower than 14
- OSFI higher or equal to 0.3 – *Group 1 and Group 2*
- OSFI lower than 0.3 – *Group 3*

3.2.4 Exclusion criteria

- Presence of dry eye symptoms preoperatively
- Occurrence of complications during surgery
- Usage of systemic antibiotic therapy
- Any pathology of the ocular surface except dry eye disease (e.g. corneal scarring, cornea ectasia)
- Ocular surgery within prior 3 months
- Ocular injury within prior 3 months
- Ocular herpes of eye or eyelid within prior 3 months
- Active ocular infection
- Active ocular inflammation or history of chronic, recurrent ocular inflammation within prior 3 months
- Eyelid abnormalities that affect lid function
- Ocular surface abnormality that may compromise corneal integrity
- Pregnancy

3.3 Study protocol

The study will be performed in the Hanusch Hospital and the connected health centres of the ÖGK, Vienna.

3.3.1 Study Visits

After baseline examination (prior to cataract extraction) three postoperative follow up visits will be scheduled (one week, one month, three month). Occurrence of dry eye signs and symptoms will be evaluated during each study visit.

3.3.1.1 Study schedule

- Visit 1 - Screening
- Visit 2 - Baseline (prior to cataract surgery, within 30 days after screening)
- Visit 3 - Follow up 1 – (7±1 days after cataract surgery)
- Visit 4 - Follow up 2 – (30±7 days after cataract surgery)
- Visit 5 - Follow up 3 – (90±7 days after cataract surgery)

3.3.1.2 Dry eye related examination

Dry eye related examination will be performed during baseline visit and during all follow ups by a masked investigator.

<u>Dry eye related examinations include all of the following</u>

- Ocular surface disease index (OSDI) score
- Photograph of the anterior segment
- Meibomian gland expressibility
- Fluorescein Staining / Lissamin green staining grading using the modified Oxford Scale
- Break Up Time (BUT)
  - Fluorescein BUT
  - Non-invasive BUT

3.3.1.3 Safety parameters

In addition to the dry eye related examination visual acuity, intraocular pressure measurements and slitlamp biomicroscopy are performed during each study visit.

3.3.1.4 Randomization

The eye scheduled for cataract surgery will be selected as the study eye. Patients included into the study are allocated to one out of three groups. Patients with an OSFI score equal or higher than 0.3 (high risk) will be randomized into group 1 or 2, using an online randomisation tool (https://randomizer.org, list randomizer) by an unmasked member of the study staff.

Group 1 receives the routine postoperative treatment (*Yellox eye drops* bid for four weeks after surgery, Bausch and Lomb, Germany), serves as the **standard of care group**.

Group 2 is the treatment group and will receive *Systane Hydration MDPF eye drops* (Alcon, USA) in both eyes four times a day starting after surgery until the three-month visit in addition to the routine treatment (*Yellox eye drops bid* for four weeks after surgery, Bausch and Lomb, Germany). Allocation to group 1 and group 2 will be randomized. All patients will receive a log to document the use of eye drops. Group 2 serves as the **treatment group**.

Patients with OSFI score below 0.3 (low risk) will be included in **group 3** and serve as a **reference group**. All patients are asked not to use any other eye drops other than their routine therapy (*Yellox eye drops bid* for four weeks after surgery, Bausch and Lomb, Germany). In case of significant complaints due to dry eye a rescue medication will be offered to the patient.

Group 1 (Standard of care): OSFI higher or equal 0.3, *Yellox eye drops* twice a day for 4 weeks

Group 2 (Treatment): OSFI higher or equal 0.3, *Yellox eye drops* twice a day

for 4 weeks and *Systane Hydration MDPF* eye drops 4 times a day for the whole study period (3 month)

Group 3 (Reference): OSFI lower 0.3, *Yellox eye drops* twice a day for 4 weeks

3.3.1.5 Withdrawal and replacement of subjects

Subjects must be withdrawn under the following circumstances:

- at their own request
- use of forbidden medication
- development of an Exclusion Criterion during study participation

3.4 Variables and biometric methods

3.4.1 Outcome variables

Primary Endpoint: Rate of dry eye symptoms defined by OSDI ≥13 at 3 months

Secondary Endpoints: Rate of DED defined by OSDI ≥13 plus at least one of the following: Presence of ocular surface staining, TFBUT <10s at 1 week, 1 and 3 months
Comparison of dry eye related parameters between the groups: TFBUT, Staining scores (modified Oxford Grading Scale) at 1 week, 1 and 3 months
Change of dry eye related parameters during the study period (baseline, 1 week, 1 and 3 months) in the study eye: TFBUT, Staining scores (modified Oxford Grading Scale)
Change of OSDI score during the study period (baseline, 1 week, and 3 months)

Exploratory Endpoints: Comparison of dry eye symptoms rate DED rate between Group 1, Group 2 and Group 3, BCVA, IOP, Concomitant medication and Adverse Events

3.4.2 Biometric methods

3.4.2.1 Data handling procedures

A case report form will be completed for each patient.

3.4.2.2 Biometric methods - outcome variables

For statistical analysis SPSS (Version 25.0) will be used. For missing data, observations will be excluded from analysis.
Descriptive data will be shown as mean, standard deviation and range. Difference in dry eye related parameters (TFBUT, Meibomian gland expressibility and staining scores) will be compared between the groups using unpaired t-tests. ANOVA for repeated measures is used to investigate changes of dry eye related parameters and OSDI Score between the study visits. Tukeys post-hoc analysis will be used for group wise comparison.

Die deskriptiven Daten werden als Mittelwert, Standardabweichung und Bereich angegeben. Die Unterschiede in den Parametern des trockenen Auges (TFBUT, Meibom-Drüsen-Expressibilität und Staining-Scores) werden zwischen den Gruppen mittels ungepaarter t-Tests verglichen. Die ANOVA für wiederholte Messungen wird verwendet, um die Veränderungen der Parameter des trockenen Auges und des OSDI-Scores zwischen den Studienbesuchen zu untersuchen. Tukeys Post-hoc-Analyse wird für den gruppenweisen Vergleich verwendet.

3.4.2.3 Biometric methods - Adverse events/Safety investigations

All adverse events will be properly listed, and an appropriate method will be used to summarise the data. Visual acuity, intraocular pressure and anterior segment findings (such as inflammatory reactions) are documented during each study visit.

3.4.2.4 Use of study findings

The findings of this study will be published by the investigators in a scientific journal (JCRS) and presented at scientific meetings (Austrian national congress 2022, DOG 2022, ESCRS 2022).

4 Methods of evaluation

4.1 Best-corrected visual acuity

Best corrected visual acuity will be assessed using ETDRS charts.

4.2 Ocular surface disease index – OSDI

The OSDI Questionnaire is a standardized questionnaire to evaluate the subjective disease burden caused by dry eye syndrome. The patients answer up to 12 questions regarding their symptoms. Thereafter, the OSDI Score is calculated.

4.3 Photography of the anterior segment

Three photographs of the anterior segment of the eye will be taken. One centered on the cornea (with and without fluoresceine) and one on the upper and the lower eyelid respectively. Images will be used to document changes on the ocular surface and eye lid margin.

4.4 OSFI Score from Villani et al.[1]

Table 4. Final Ocular Surface Frailty Index Composition

Item	Results	Points
Connective tissue diseases	No	0
	Yes	1
Thyroid malfunction	No	0
	Yes	1
Psychiatric conditions*	No	0
	Yes	1
Computer use†	No	0
	Yes	1
Ocular allergy	No	0
	Yes	1
History of refractive surgery	No	0
	Yes	1
Topical drugs‡	No	0
	Yes	1
TBUT with fluorescein (sec)	≥10 s	0
	5-9 s	0.50
	0-4 s	1
Meibomian gland expressibility (digital expression)	Grade 0: clear meibum easily expressed	0
	Grade 1: cloudy meibum expressed with mild pressure	0.33
	Grade 2: cloudy meibum expressed with moderate pressure	0.66
	Grade 3: meibum not expressed with more than moderate pressure	1
LIPCOF	Grade 0	0 points
	Grade 1	0.33 point
	Grade 2	0.66 point
	Grade 3	1 point

BAK = Benzalkonium chloride; LIPCOF = lid parallel conjunctival folds; TBUT = tear film breakup time.
*Including affective disorders, somatoform disorders, anxiety, and depression.
†Exposure >4 hours/day.
‡Current use of at least 1 of the following topical drugs: antiglaucomatous, antiallergic, antiviral, decongestants, miotics, mydriatics, nonsteroidal anti-inflammatory, or at least 3 drops/day BAK preserved.

4.5 Slit lamp biomicroscopy

Conventional slitlamp is used to investigate anterior segment of the eye and the eyelids.

4.6 Fluorescein Staining / Lissamin green staining grading using the Oxford Scale

Fluorescein and Lissamin green dye will be used to examine the anterior surface of the human eye. Anterior segment photography will be used to document the findings, which are graded using the modified Oxford Grading Scale.

4.7 Break Up Time (BUT)

Fluorescein Break Up time is measured after instillation of 1ul fluorescein into the inferior fornix. Using a stopwatch time after the last blink until the first break up of the tear film is recorded. Three consecutive measurements are performed, and the average is calculated.

Non-invasive BUT is measured using the Dry eye module build into the Sirius device (CSO) and MS – 39 (CSO).

5 Schedule of Events

**Visit name**	**VISIT 1**	**VISIT 2**	**VISIT 3**	**VISIT 4**	**Visit 5**
**Visit date**		**Day 0**	**Day 7**	**Month 1**	**Month 3**
**Visit Description**	Screening	Baseline	Follow up 1	Follow up 2	Follow up 3
**Visit date window**	-30d to 0	Day 0	Day7 ± 1	Day 30 ± 7	Day 90 ± 7
Informed consent	x				
Demographic data[a]	x				
Medical history	x				
Prior medication[b]	x				
Eligibility check	x				
Urine pregnancy test[c]	x				
BCVA (ETDRS Chart)	x	x	x	x	x
Intra ocular pressure	x	x	x	x	x
Slit Lamp Examination	x	x	x	x	x
Randomization	x				
OSDI score	x	x	x	x	x
OSFI score	x				
DEQ 5	x				
Meibography	x				
Photograph of the anterior segment		x	x	x	x
Fluorescein/Lissamin green staining	x	x	x	x	x
Meibum expressibility	x	x	x	x	x
Break up time	x	x	x	x	x
Concomitant medication[d]		x	x	x	x
Adverse event collection[e]		x	x	x	x
Cataract surgery		x			

a…..Age, gender, race

b…..until randomization

c…..in women of childbearing potential only

d…..as of randomization

e…..as of informed consent signature

6 Timeline

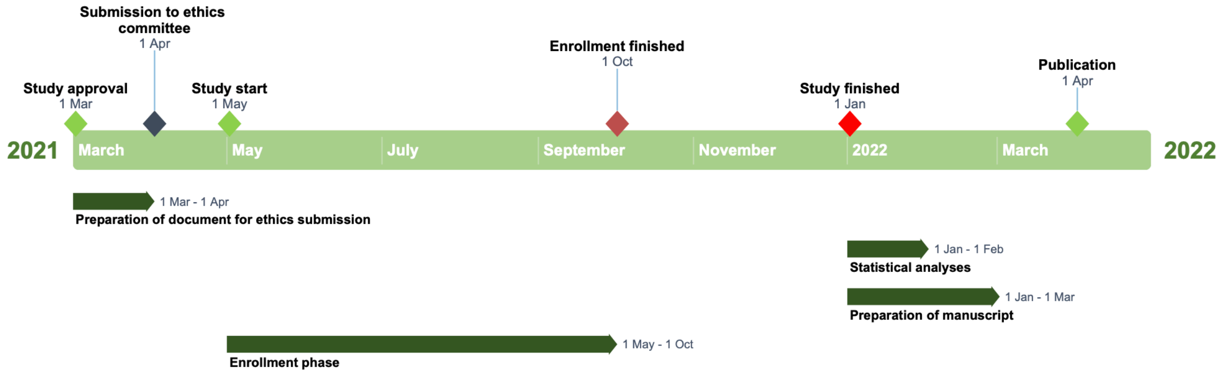

7 References

1 Villani E, Marelli L, Bonsignore F, Lucentini S, Luccarelli S, Sacchi M, et al.: The Ocular Surface Frailty Index as a Predictor of Ocular Surface Symptom Onset after Cataract Surgery. Ophthalmology 2020; DOI: 10.1016/j.ophtha.2019.12.012

2 Mrukwa-Kominek E, Baranska K, Jadczyk K: First clinical reports on the application of the modern dual-polymer formula in aqueous deficiency dry eye syndrome: Polish observations; in : ESCRS Winter Meeting. 2016.

3. Eric Carlson, et al. Impact of Hyaluronic Acid-Containing Artificial Tear Products on Reepithelialization in an In Vivo Corneal Wound Model. JOURNAL OF OCULAR PHARMACOLOGY AND THERAPEUTICS Volume 34, Number 4, 2018 Mary Ann Liebert, Inc. DOI: 10.1089/jop.2017.0080
